# The Molecular Mechanism of Retina Light Injury Focusing on Damage from Short Wavelength Light

**DOI:** 10.1155/2022/8482149

**Published:** 2022-04-19

**Authors:** Bin Fan, ChunXia Zhang, Jing Chi, Yang Liang, XiaoLi Bao, YunYi Cong, Bo Yu, Xun Li, Guang-Yu Li

**Affiliations:** ^1^Ophthalmology Department, Second Hospital of Jilin University, Changchun 130000, China; ^2^Jilin Provincial Institute of Education, Changchun 130022, China

## Abstract

Natural visible light is an electromagnetic wave composed of a spectrum of monochromatic wavelengths, each with a characteristic color. Photons are the basic units of light, and their wavelength correlates to the energy of light; short-wavelength photons carry high energy. The retina is a fragile neuronal tissue that senses light and generates visual signals conducted to the brain. However, excessive and intensive light exposure will cause retinal light damage. Within the visible spectrum, short-wavelength light, such as blue light, carries higher energy, and thus the retinal injury, is more significant when exposed to these wavelengths. The damage mechanism triggered by different short-wavelength light varies due to photons carrying different energy and being absorbed by different photosensitive molecules in the retinal neurons. However, photooxidation might be a common molecular step to initiate cell death. Herein, we summarize the historical understanding of light, the key molecular steps related to retinal light injury, and the death pathways of photoreceptors to further decipher the molecular mechanism of retinal light injury and explore potential neuroprotective strategies.

## 1. Introduction

Light has laid the foundation for the growth and prosperity of all life on Earth by triggering various chemical, biological, and physiological reactions. In addition, light also acts as a primary tool when perceiving the world and aids in forming visual perception for all the living things possessing visual potential. The retina is the only fragile neuronal tissue that can sense light and produce visual signals to the brain. However, excessive and intensive light irradiation can lead to retinal light damage [1, 2]. The retinal injury model for exposure to white light has been widely used in experimental research [3–7]. White light is comprised of a superposition of monochromatic wavelengths in the visible light spectrum. The severity and mechanism of retinal injury induced by different monochromatic light waves may vary considerably [6, 8, 9]. Many studies show that visible light primarily induces retinal damage through photochemical mechanisms [10, 11]. The term “action spectrum” was explicitly proposed to describe the efficiency of the photochemical reaction produced by electromagnetic radiation, which is correlated with radiation wavelengths. In research, the efficiency of the photochemical reaction is often evaluated in various ways, such as the specific decrease in a-wave or b-wave amplitude of electroretinogram, visible changes of retina fundus, or the changes of specific retinal structures observed with light or electronic microscope [12]. Photons are the basic units of light. According to Planck's formula, the wavelength of photons determines the energy level it carries. The shorter wavelength, the higher the energy that photons are with [13]. For different monochromatic light, including ultraviolet radiation, the sensitivity of retinal light injury gradually decreases from short wavelength to long wavelength based on the action spectrum.

This paper mainly focuses on short-wavelength light and summarizes the historical understanding of light essence, the key molecular steps in the process of visual formation related to retinal light injury, and the death pathways of photoreceptors to further decipher the molecular mechanism of retinal light injury and to explore potential neuroprotective strategies.

## 2. The Nature of Light

The debate about the nature of light has been going on for centuries in the field of Physics. Before the seventeenth century, two main incompatible theories had been competing with each other. Huygens first proposed the undulatory theory of light in 1690: light transmits in the form of waves, and the light waves spread in all directions from the light source and are perceived by the human eye. However, Isaac Newton, who performed his optical experiment in 1666, favored the corpuscular theory: Light is a particle, and the light ray emits as tiny particles or “corpuscles” from the light source, passing through the space like a bullet to reach the human eye. Until the beginning of the nineteenth century, Thomas Young demonstrated the interference properties of light through a double-slit experiment, in which two overlapping beams of light produced bright and dark bands, analogous to the interference patterns generated by the superposition water waves. Therefore, the corpuscular theory of light was challenged by the undulatory theory. However, by the 1830s, Michael Faraday first proved the relationship between electric and magnetic fields. Later on, James Clerk Maxwell used Faraday's field theory to merge them into a single concept mathematically—the electromagnetic wave, simultaneous with transverse electric and magnetic components. The calculated electromagnetic waves will be about 300,000 kilometers/sec at a speed similar to light. In 1900, Max Planck hypothesized that the electromagnetic spectrum from red hot bodies could be interpreted only when radiation is emitted or absorbed in “quanta.” Thus, the theory of Planck and Einstein became the cornerstone of quantum optics in the twentieth century. After 1905, with the advent of the Special Theory of Relativity, Einstein proposed a more modern form of Newton's corpuscular theory. The emission and absorption of light are not only quantized (formed into quanta) but light itself is also quantized [14, 15].

Today, physicists believe that light has wave-particle duality [13] and more importantly, light is a form of energy transmission. In the narrow sense, light is an electromagnetic wave that human eyes can see, defining it as the visible light spectrum. In the scientific interpretation, light refers to the entire electromagnetic spectrum. Electromagnetic radiation occurs over an extensive range of wavelengths, from gamma rays with a wavelength of less than 1 × 10^−11^ meters to radio waves measured in meters. According to modern quantum optics theory, light is regarded as electromagnetic waves and a beam of particles called photons traveling at the speed of light *C* in a vacuum. These particles should not be simply regarded as classic billiard balls but particles described by wave functions in a limited range in quantum optics. Each photon is a quantum of electromagnetic energy regarded as a discrete particle with zero mass, no charge, and infinite life. When a photon hits an object, the object absorbs the photon energy to excite the ground state electrons in the molecule, in which excited electrons jump to a higher energy level. When the excited state electron decays from a high-energy level to a low-energy level, the molecule emits light in the form of a specific wavelength of photons carrying a specific quantum of energy [16].

## 3. Visible Light and Colors

The electromagnetic waves that the human eye can perceive are called visible light, and the visible spectrum ranges from about 400 nanometers to 700 nanometers [17]. Other creatures may perceive light beyond this range, but human eyes cannot see it. Different wavelengths of light form various colors after being perceived by the human eye. Commonly, colors can be divided into red, orange, yellow, green, blue, indigo, and violet based on visual experience. The light with different narrow bands corresponding to colors is called monochromatic light, and the wavelength range of monochromatic light is often set as purple (380–450 nm), blue (450–495 nm), green (495–570 nm), yellow (570–590 nm), orange (590–620 nm), or red (620–750 nm). However, a few people can see further into ultraviolet and infrared [18, 19], so the wavelength edge of visible light is not well-defined. The light that the human eye can perceive not only needs to be in the visible spectrum but also requires sufficient energy for photons to interact with the photosensitive groups in the retinal photoreceptor cells, change the molecular conformation, and then induce the action potential of the photoreceptor cells and form nerve impulses to further conduct to the visual cortex of the brain [20, 21]. Therefore, the human eye can perceive the color of light and perceive the brightness of the light.

**Figure 1:**
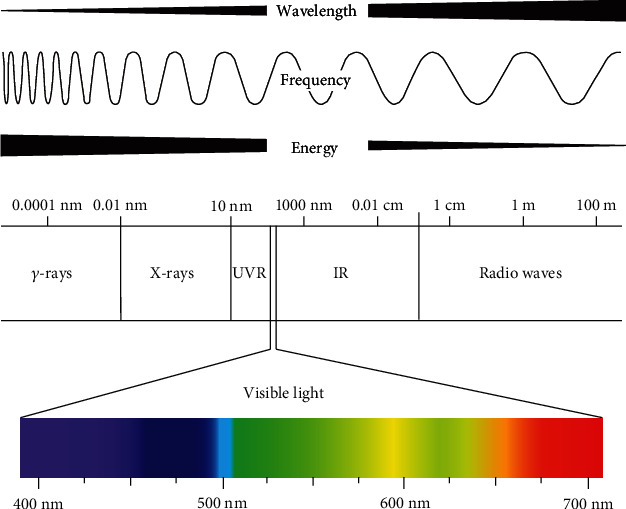
The relationship between photon energy and wavelength. According to the energy equation of quantum mechanics, *E* = *h* × *C*/*λ*, it is easy to find that the shorter wavelength photon has, the higher energy it carries. Therefore, the energy of *γ*-rays is the greatest, while radio waves energy is the least. Among visible light, violet light has the shortest wavelength, highest frequency, and energy, and red light has the longest wavelength, shortest frequency, and lowest energy (illustrated by Dr. Bo Yu).

Different wavelengths of photons possess different energy. According to the energy equation of quantum mechanics, *Energy* (*E*) = *h* × *C*/*λ*, where energy *E* = bandgap, *h* = 6.626 × 10^−34^ Joule second (Planck's constant), *C* = 2.99 × 108 m/sec (speed of light), and *λ* = absorption peak (wavelength) [22]. From this formula, it is easy to find that the energy of a photon is proportional to its frequency: high-frequency light has high energy; low-frequency light has low energy. Therefore, the energy of gamma rays is the greatest, while the energy of radio waves is the least. Among visible light, violet light has the shortest wavelength, which has the highest frequency and energy. Red has the longest wavelength, shortest frequency, and lowest energy (Figure 1). The energy of a single visible light photon is minimal, about 4 × 10^−19^ joules. For convenience, the unit of energy is usually converted to electron volts (eV). One electron volt is equal to the energy obtained when the electric potential changes by 1 volt: 1 eV = 1.6 × 10^−19^ joules. The energy of a single photon in the visible light spectrum ranges from about 1.8 eV (red light) to about 3.1 eV (purple light). The human eye cannot detect individual photons.

## 4. Phototransduction and Visual Cycle

The eye's refractive system is composed of the transparent cornea, aqueous humor, lens, and vitreous. Light enters the eye and focuses on the retina to form a clear image via the refractive system. The retina mainly contains five types of neuronal cells. When light acts on the retina, it must pass through all neuron cell layers to reach and activate the photoreceptor cells in the outermost layer of retina. The photoreceptor cells are a specialized type of neuroepithelial cell capable of absorbing light and converting it into an electrical signal, which is then transmitted through the bipolar cells to the inner ganglion cells [23]. Horizontal cells and amacrine cells are responsible for the lateral communication between neurons [24].

There are two types of photoreceptors in the retina: rods and cones. The rod is responsible for vision under weak light conditions, while the cone cells are responsible for vision under bright conditions. In addition, the cone cells participate in the formation of color vision and can be further divided into three types of cone cells, L, M, and S, according to the response wavelength ranges of red, green, and blue, respectively [25]. Photoreceptor cells only have axons, no dendrites, and form a specialized disc-shaped outer segment containing photopigments. The photopigments that absorb light have similar structures, consisting of a protein called opsin and a small attached molecule known as the chromophore. The chromophore absorbs photons of light, using a mechanism that involves a change in its configuration [26–28].

Phototransduction is the process by which light becomes an electrical signal through photoreceptor cells, which occurs inside the photoreceptor cells. Unlike normal neurons, light stimulating photoreceptor cells can lead to hyperpolarization rather than depolarization [28]. The membrane potential of photoreceptor cells receiving light will decrease, while unstimulated photoreceptor cells are naturally depolarized. In general, the resting membrane potential of neurons is approximately −65 mV, while the membrane potential of photoreceptor cells in a dark environment is −40 mV [29]. This phenomenon is called dark current. The second messenger, cGMP (cyclic guanosine monophosphate), indirectly regulates the dark current by opening sodium channels and calcium channels on the photoreceptor cell membrane [30–32]. Light stimulating photoreceptor cells will cause the decreased concentration of cGMP and the increased membrane potential up to −65 mV for rod cells, forming hyperpolarization, which is contrary to the depolarization behavior of normal neurons after being stimulated [28].

The photosensitive pigment in the outer segment of the photoreceptor cell is called rhodopsin, which is a covalent complex formed by the combination of opsin and retinal. Light absorption causes a double bond of 11-cis-retinal to flip and form its isomer all-trans retinal. The isomers of retinal convert rhodopsin into metarhodopsin II [33, 34]. The activated metarhodopsin II diffuses on the cell membrane and interacts with transducin to release GDP (guanosine diphosphate), which binds to GTP (guanosine triphosphate). The transduction protein is divided into three subunits (subunit), *α*, *β*, and *γ*, and GTP separates the *α* unit from the other two to form T*α*-GTP. The T*α*-GTP forms a complex with PDE, another protein on the cell membrane, to catalyze the hydrolysis of cGMP to 5′-GMP (5′-guanylic acid) [35]. As mentioned earlier, this process eventually causes the Na^+^ and Ca^2+^ ion channels to close, causing photoreceptor cell hyperpolarization.

One of the critical features of this complex biochemical cascade triggered by photon capture is that it provides an enormous signal amplification [36]. It is estimated that a single photon-activated rhodopsin molecule can activate 800 transduction protein molecules, which is about 8% of the surface molecules of the disk. Although each transduction protein molecule activates only one phosphodiesterase molecule, each of these molecules can catalyze the breakdown of up to six cGMP molecules [37]. As a result, the absorption of a single photon by rhodopsin molecules causes approximately 200 ion channels to close, or approximately 2% of the number opened in the dark in each rod. This amount of channel closure results in a net change in membrane potential of approximately 1 mV [38].

The visual cycle includes a series of enzymatic reactions in photoreceptors and retinal pigment epithelial cells (RPE) to regenerate the visual rhodopsin chromophore, 11-cis-RAL. The photoisomerization of 11-cis-RAL chromophores of rhodopsin into all-trans retinal leads to a series of complex metabolic transformations, which eventually leads to light perception [39, 40]. There are various mechanisms inside the photoreceptor cells for negative feedback regulation to prevent oversensitivity in a high-brightness environment. These negative feedback mechanisms limit the duration of this amplification cascade and restore various molecules to their inactive state. For example, inhibitory proteins block the ability of activated rhodopsin to activate transduction proteins and promote the breakdown of activated rhodopsin [41]. The all-trans-RAL is then separated from the opsin, diffuses into the cytosol of the outer segment, and is transported out of the outer segment and into the pigment epithelium, where it is finally converted into 11-cis-RAL by suitable enzymes. After being transported back to the outer segment, the 11-cis-RAL recombines with the opsin in the receptor disc. The recycling of rhodopsin is essential to maintain the light sensitivity of photoreceptors [39, 42]. In addition, after light stimulation, photoreceptors not only need to terminate PDE hydrolysis of cGMP but also need to quickly restore the dark level of cGMP, which is determined by PDE-mediated hydrolysis and guanylate cyclase (GC)-mediated synthesis [43]. GC activity is regulated by Ca^2+^, which is mediated by guanylate cyclase activating proteins (GCAPS). GCAPS belong to a large family of calmodulin-like Ca^2+^-binding proteins [44]. In the dark, high Ca^2+^ concentrations promote the formation of the Ca^2+^-binding form of GCAPS, thus inhibiting GC. During the photoreaction, when the Ca^2+^ concentration decreases, the dissociation of Ca^2+^ causes GCAPS to activate GC, which quickly restores the basic cGMP concentration. This regulation is the most important negative feedback mechanism triggered by Ca^2+^ in high-light environments, and it maintains the homeostasis of light conduction in photoreceptor cells [35, 45, 46].

The role of the visual cycle in the molecular mechanism of light-induced retinal degeneration is also well manifested. The photooxidation of all-trans-RAL is considered to be a possible sensitizer of retinal light damage. Excessive accumulation of all-trans-RAL may also lead to retinal photochemical damage [47]. Two all-trans-RAL and phosphatidylethanolamine molecules can be converted into A2E [48]. Recent studies show that ultraviolet or blue light irradiation can mediate phototoxicity through A2E in the retina [49, 50].

## 5. Action Spectrum of Retina Damage

Before the first half of the twentieth century, it was thought that light caused retinal damage through thermal mechanisms. However, in 1966, Noell et al. found that rats exposed to green light for up to 2 days showed extensive functional and histological damage, which the energy of the exposed light was much lower than that level results in photothermal damage; thus, this nonthermal mechanism is called retinal photochemical damage [51].

It is well known that prolonged exposure to intense light causes photochemical damage to the retina. The retina light injury was found to be related to two kinds of action spectrum. In rats, Organisciak et al. first found that the light spectrum inducing photochemical damage to the retina matches the absorption light spectrum of rhodopsin, which is called the rhodopsin absorption spectrum (RAS) [52]. However, Ham et al. found in macaques that as the wavelength of visible light goes from long to short (from red to ultraviolet light), the retina sensitivity to photochemical damage increases gradually [53]. Therefore, this phenomenon is called short-wavelength action spectrum (SAS), but later on, this spectrum was also confirmed on rats with further research [54–56].

The action spectrum is described as a formula interpreting the relationship between the lowest energy and wavelength of electromagnetic radiation that induces photochemical damage to the retina. Usually, the photochemical damage of the retina can be assessed functionally and structurally, such as determining the value of a-wave or b-wave of the electroretinogram (ERG) or detecting structural changes of the retina with a light or electron microscope [57]. A growing number of studies also use OCT or OCTA to assess the changes in retinal structure induced by light damage because OCT and OCTA can provide high-resolution images of different levels of the retina and continuous changes in damage on living animals [58, 59].

Gorgels et al. determined the threshold dose of photochemical damage to the retina caused by monochromatic light of different wavelengths [60]. In rats, macaques, and other animals, it was found that the shorter the wavelength of monochromatic light, the lower the threshold energy of photochemical damage to the retina, while the longer wavelength of monochromatic light has higher threshold energy. Li et al. have shown that UV damage to the lens of macaque eyes is immediately apparent and different wavelengths can cause different types of damage [61]. Ultraviolet rays cause extensive damage to photoreceptors, depigmentation, and swelling of RPE with many inclusions [62].

Wu et al. demonstrated that after Sprague Dawley rats were exposed to diffuse blue light of 0.64 W/m^2^ (400–480 nm), photoreceptor cells showed typical apoptotic characteristics, including progressive concentration and marginalization of chromatin, nuclear contraction or curl and fragmentation, cytoplasmic concentration, formation of apoptotic bodies, and rapid clearance of dead cells in damaged areas. After illumination, TUNEL positive nuclei dispersed in the outer nuclear layer. Many TUNEL positively labeled photoreceptors peaked between 8 and 16 hours and decreased 24 hours after illumination [63]. TUNEL positive cells were mainly distributed in the upper temporal region of the retina, the most sensitive region to blue light injury. R91w-NRL^−/−^ double mutant mice showed ordered full cone retina, normal retinal vascular system, and strong bright vision function. Geiger et al. found that after NRL^−/−^ double mutant mice were exposed to blue light, a large number of nuclear pyknosis occurred in the outer retinal nuclear layer, photoreceptors died, and the blood-retinal barrier in the retina was damaged [64]. Javier et al. revealed that exposure of high-intensity (5000 lux) white light to young albino mice for seven days significantly induced retinal light damage; after filtering out the blue light component significantly reduced the retinal light damage even under exposure to intense irradiation. These results suggest that short-wavelength light, such as blue light, is the major risk component in the visible light spectrum inducing retinal light injury [65].

Although there is little literature on direct experimental evidence of retinal damage from light exposure in the human eye, there is considerable indirect evidence to show that excessive light exposure can cause acute and chronic damage to the retina. Sharma reported a series of cases among people who lived at high altitudes during the winter months, all of whom had a loss of central vision typical of prolonged sunbathing. Optical coherence tomography shows that the outer retinal defect in all patients involves the ellipsoidal region of solar retinopathy. Prolonged excessive solar radiation leads to acute fovea injury, resulting in lateral retinal defects [66] . In the human retina, documented solar radiation damage ranges from the acute effects of staring at the sun to injuries caused by prolonged exposure to a bright environment. The lesion occurs in the same region of degeneration in age-related macular degeneration (AMD) [66]. There have also been many reports that short wavelengths of light, such as ultraviolet and blue light, can significantly lead to a decrease in cell activity in human-derived RPEs cultured in vitro and induce an increase in cell death [67–69].

**Figure 2:**
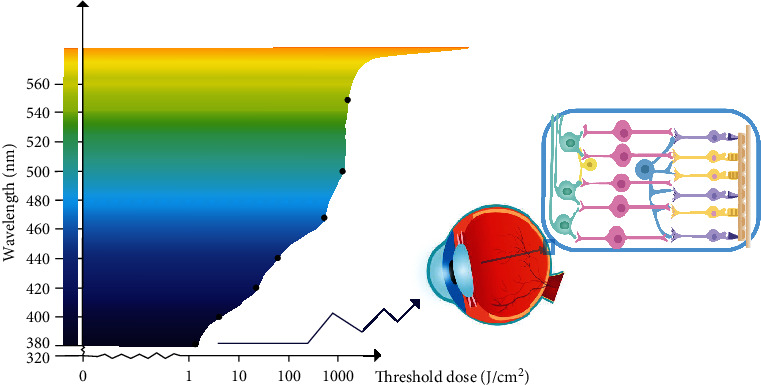
Action spectrum. As the wavelength of visible light goes from long to short, the threshold doses for retinal light damage decrease, and the retinal sensitivity to photochemical damage increases gradually (illustrated by Dr. YunYi Cong).

Further research indicates on photochemical damage to the retina is reduced as the wavelength of light increases. A few studies have shown that long-wavelength visible light, such as green light, tends to cause photothermal damage in the retina by increasing the energy of light to a higher level, while the research of photochemical damage induced by other, longer wavelength visible lights, such as yellow light or red light, is rare [70, 71]. Therefore, the following discussion on the molecular mechanism of retinal photochemical damage mainly focuses on blue light and ultraviolet radiation (Figure 2).

## 6. Photooxidation an Initial Step Triggering Death Cascade

In addition to assisting in the formation of vision, light can also perform biological modulation on the eye, but excessive light exposure can damage the retina. Previous studies have shown that moderate outdoor activities can prevent the formation and control the progression of myopia in adolescents and its mechanism may be related to reducing long-term nearsightedness and increasing the contraction and relaxation activities of ciliary muscle [72, 73]. It has also been reported that long-term exposure to short wavelength light, such as blue light, can induce hyperopia in animals. This is because the image formed by short-wavelength light is focused in front of the retina, and in order to see the image more clearly, the brain sends regulatory signals to promote scleral remodeling and shorten the ocular axis, causing hyperopic changes [74, 75]. Recent multicenter clinical studies have shown that short-term intermittent stimulation of the retina with low-energy red laser (650 nm, 2 MW) in adolescents can effectively prevent the progression of myopia and shorten the ocular axis, but the therapeutic molecular mechanism of low-energy red light in the prevention and control of myopia needs to be further studied [76]. However, there are also many reports that long-term excessive exposure to sunlight, especially short-wavelength light such as blue light and ultraviolet light, can cause retinal photochemical damage, which is closely related to the pathogenesis of AMD [77]. Because the energy of a photon varies with its wavelength and because photosensitive molecules absorb different photons, the exact mechanisms underlying retinal photochemical damage caused by different monochromatic lights are assumed to be different [78]. Here, we will focus on the two most common kinds of short-wavelength light, UV and blue light, to determine the molecular mechanisms underlying photochemical damage.

### 6.1. Mechanism Underlying UV Light-Induced Photochemical Damage

Ultraviolet rays (UVR) produced by the sun can be divided into three categories: UVA, UVB, and UVC [79]. UVA has the longest wavelength, ranging from 320 to 400 nm with a penetration rate through ozone of about 95% [80]; UVB ranges from 290 to 320 nm, and its penetration rate is 5%; and UVC ranges from 200–290 nm and can be completely absorbed by the Earth's stratosphere [80]. Although the eye consists of a cornea, lens, and other protective barriers that can absorb most UVA and UVB, a small amount of ultraviolet radiation can still reach the retina [81], especially in the eyes of children under the age of ten. However, because the number of chromophores increases with age, the transmittance of ultraviolet radiation decreases [82]. In addition, if the intraocular lens cannot effectively filter these ultraviolet rays for patients who have undergone cataract surgery, there will be more retinal damage [83].

Since there are spectrum absorption peaks of ultraviolet light for proteins and DNA in retinal neuronal cells, the photon energy of ultraviolet light can directly transfer to these macromolecules and mediate photochemical reactions. Ultraviolet radiation-induced damage can be through two mechanisms:

- Cellular macromolecules directly absorb the energy of UV light, resulting in the formation of excited states and subsequent chemical reactions. For example, nucleic acid bases of DNA may form into dimers after directly absorbing UV energy, including cyclobutane pyrimidine species, pyrimidine-pyrimidinone compounds, and their Dewar isomers. These three types of dimers are closely related to the mutagenicity of ultraviolet radiation, and their typical characteristics are with high levels of CC-->TT and C-->T crosses [84]
- Photooxidation mechanism: the energy of the ultraviolet photons is absorbed by the photosensitizers in the cell, and the activated photosensitizers can further induce oxidative stress damage. (I) Oxygen free radicals are generated during electron transfer and hydrogen extraction; (II) energy transfers to O^2^ to induce singlet reactive oxygen species. The main product of singlet active O^2^ induced DNA damage is 8-oxocitrulline, a common type of damaged DNA produced by a series of oxidants induced by UVR. In addition, UV-induced protein damage can also be mediated by singlet O^2^, which can react with Trp, His, Tyr, Met, Cys, and cystine side chains. The initial products might be aromatic residues, endoperoxides, and zwitterions with sulfur residues that can serve as intermediaries, further undergoing a multi-step reaction, leading to a significant increase in protein cross-linking and aggregation, and functional inactivation, but almost no protein fragments are produced [85]

### 6.2. The Mechanism of Blue Light-Induced Photochemical Damage

As a short-wavelength visible light, blue light is generally defined with a wavelength ranging from 400 to 500 nm [86]. Due to the intraocular refractive system filtering effect, the potentially damaging UVR reaching the retina is limited. However, if the wavelength of light exceeds 400 nm, the radiation transmittance through the refractive system will be significantly increased. In children, more than 65% of 460 nm blue light might reach the retina. With age, lens proteins, such as crystallin, become progressively less transparent, resulting in a relative decrease in the transmittance of visible light in the blue region of the spectrum [87]. According to Planck's equation, the wavelength is inversely proportional to the energy of the photon [88]. Therefore, blue light is characterized by short wavelength with high energy relative to other monochromatic lights [89], rendering it capable of causing severe damage to the retina. When the retina is exposed to excessive blue light, retinal phototoxicity or blue light hazard occurs, usually due to photochemical damage depending on the total dose received by the retina as a function of the irradiance intensity and exposure duration.

Blue light induces photochemical damage to the retina mainly through a photooxidation reaction [90]. Many endogenous chromophores can absorb blue light energy in photoreceptor cells to further trigger photochemical damage, breaking bonds in certain molecules through direct electron exchange or hydrogen exchange, resulting in reactive oxygen species (ROS) [91]. The transfer of electrons from the excited state of the photosensitizer to oxygen can produce singlet oxygen (1O^2^), and subsequent reactions may produce superoxide radicals (O^2•-^), hydrogen peroxide (H_2_O_2_), and hydroxyl radicals (•OH) [92]. However, excessive intracellular ROS is fatal to cells; it can act on polyunsaturated fatty acids in the cell membrane structure, causing lipid peroxidation and destroying the cell membrane structure. In addition, excessive ROS also can penetrate the nuclear membrane to cause genetic mutations or act on intracellular proteins leading to enzyme and other protein dysfunction [93]. Many studies have shown that blue light exposure may cause a significant increase in ROS production in the retina [4, 94–96].

In addition, photoreceptors are rich in mitochondria. The various chromophores in the mitochondria have an absorption peak at the wavelength of blue light (400–500 nm), enabling the mitochondria to absorb more blue light energy and further inducing photochemical damage [97]. Therefore, excessive exposure to blue light can predominantly cause ROS accumulation and oxidative stress damage, affecting the structure and function of retinal mitochondria and triggering mitochondrial-related cell death.

## 7. The Death Pathways of Photoreceptors

### 7.1. Parthanatos

Parthanatos (from Greek death Thanatos) is a programmed death caused by excessive activation of poly(ADP-ribose) polymerase-1 (PARP-1) [98]. Parthanatos is a mitochondrial-related cell death paradigm independent of caspase. PARP is a cellular ribozyme responsible for repairing DNA damage. When light exposure induces nuclear DNA breaks, it quickly causes the activation of PARP-1 to synthesize PAR to repair damaged DNA. However, a large amount of PAR can induce the formation of pores in the mitochondrial outer membrane activating apoptosis-inducing factor (AIF) anchored on the mitochondrial inner membrane to translocate into the nucleus, resulting in the degradation of nuclear chromatin. In addition, excessive activation of PARP leads to intracellular NAD^+^ energy depletion, further impairing cellular metabolic processes to promote death [99–101]. Li et al. demonstrated that excessive visible light exposure led to massive activation of PARP-1 in cultured photoreceptor cells (661 W), accompanied by AIF nuclear translocation. Applying a specific PARP inhibitor or gene knockdown PARP showed a significant protective effect on photoreceptor cells under light injury [102, 103]. In addition, Ji et al. revealed that PARP inhibitor showed a significant neuroprotective effect on rat retina against light injury [104].

### 7.2. Necroptosis

Necroptosis is a typical type of regulatory necrosis characterized by necrotic cell death that depends on the activation of receptor-interacting serine/threonine-protein kinase 1 (RIP1), receptor-interacting serine/threonine-protein kinase 3 (RIP3), and mixed lineage kinase domain-like pseudokinase (MLKL). After initiating cell death, RIP1 phosphorylates and activates RIP3, which causes the activation of MLKL, forming a necrosome complex [105, 106]. The activation of necrosomes leads to oligomerization of phosphorylated MLKL on the cell membrane; MLKL oligomers possess pore-forming activity or cause the regulatory dysfunction of Na^+^ or Ca^2+^ channels leading to cell rupture and necrosis [107, 108]. The phosphorylation of MLKL and necrosome formation are currently considered to be markers of necroptosis. However, in a specific cellular environment, necroptosis proteins RIPK1, RIPK3, and MLKL can also participate in other cell pathological processes, such as inflammation [105, 108]. Therefore, to verify necroptosis, it is necessary to demonstrate the presence of necrosomes in concert with the activation of necrosis. The pharmacological or genetic inhibition of RIPK1, RIPK3, or MLKL can block programmed necrosis. Yang showed that blue light exposure (450 nm, 300 lux) caused a caspase-independent death in R28 cells (a retinal neuron), and the application of a specific blocker of necroptosis pathway, necrostatin-1, may markedly attenuate cell death induced by blue light exposure, indicating the necroptosis pathway involves the blue light-induced death of retinal neurons [109]. Jaadane et al. demonstrated that in Wistar rats with retinal light injury induced by four different wavelengths of blue light (507, 473, 467, and 449 nm) exposure, the caspase-independent apoptosis and activation of necroptosis were observed in photoreceptors. The death of photoreceptors was inversely proportional to the wavelength and excessive blue light exposure induced necrosis-like phototoxicity in photoreceptors [96].

### 7.3. Autophagy

Ashford and Porter proposed autophagy after discovering the phenomenon of “eating yourself” in cells in 1962. It refers that part of intracellular cytoplasm and organelles, proteins, and other components is sequestered by a specialized double-layer membrane to form autophagosomes, and further fuses with lysosomes to form autolysosomes [110]. The degradation of the encapsulated cellular contents may assist the metabolic needs of the cell and the renewal of some organelles. The molecular signaling mechanism of autophagy can be divided into the following stages:

(I) Autophagy induction stage: Mainly mediated by the Atg1 complex, as the activity of rapamycin target protein complex 1 (mTORC1) is inhibited, the level of phosphorylated atg13 is reduced. Then, atg13-atg1-atg17 complex is formed to induce autophagy [111]
(II) Nucleation process: vps34 forms a complex with atg6, which acts on the nucleation of membrane vesicles mediating the formation of the pre-autophagosomal structure. Later on, the aggregation of atg12-atg5 and atg16 forms polymers and promotes the extension and expansion of autophagic vesicles together with LC3 [112, 113]
(III) The extension stage of autophagy: This mainly depends on two ubiquitination-like systems:
  - The binding process of atg12: The binding process of atg12 is a ubiquitination-like process, which requires the participation of ubiquitin-activating enzymes E1 and E2. The E1-like enzyme Atg7 first activates Atg12 and then transported by the E2-like enzyme atg10 and bound to ATG5; it combines with atg16 to form a multibody complex atg12-atg5-atg16. This complex is located on the outer membrane surface of the pre-autophagy structure and participates in expanding the outer membrane of the pre-autophagy [114]
  - Modification process of LC3: The modification process of LC3 also requires the participation of ubiquitin-activating enzymes E1 and E2. After the formation of the LC3 precursor, it is processed into cytoplasmic soluble LC3-I by atg4 and then covalently connected with phosphatidylethanolamine (PE) to form lipid-soluble lc3-pe (i.e., LC3-II) under the action of the E1-like enzyme Atg7 and the E2-like enzyme atg3, participating in the extension of the membrane. LC3-II can bind to the newly formed membrane until the autophagy lysosome is formed [115]. Therefore, LC3-II is often used as a marker of autophagy and is an important multisignal transduction regulatory protein located on the vesicle membrane of autophagy. The mammalian atg12-atg5 ubiquitination process and LC3 ubiquitination process do not operate independently. They can interact and regulate each other [116]
(IV) Autophagosome maturation stage: This mainly refers to the process in which autophagosomes fuse with lysosomes to form autophagy lysosomes through a microtubule skeleton under the action of endolysosome classification complex and monomer GTPase. Lysosomal-related proteins involved in the maturation stage include lamp1, Lamp2, and UVRAG (UV resistance-related tumor suppressor gene) [117, 118].
(V) Autophagosome cleavage stage: This refers to the cleavage of the autophagy lysosomal membrane and the degradation of its contents under the action of lysosomal hydrolase. Amino acids and some proteins produced during degradation can provide nutrition, energy, or recycling for cells [119]

Xia et al. found that the expression of autophagy markers LC3, beclin-1, and p62 was upregulated in blue light-induced retinal injury, indicating that autophagy plays an essential role in response to blue light-induced retinal injury [120]. Feng et al. demonstrated that in vitro cultured RPE cells, A2E, and blue light cotreatment significantly increased the expression of the endoplasmic reticulum stress-related apoptotic molecules CHOP and caspase-12. The observation of autophagosome formation at the ultrastructural level indicates the activation of autophagy. In addition, the dot distribution of LC3 immunofluorescence and the enhanced transformation of LC3-I to LC3-II were found in A2E and blue light-treated RPE. Furthermore, the inhibition of autophagy makes RPE more vulnerable to A2E and blue light damage.

On the contrary, rapamycin, an autophagy inducer, alleviates endoplasmic reticulum stress and promotes RPE survival [69]. Autophagy may be a critical endogenous cytoprotective process to reduce RPE damage caused by blue light exposure. Using the Drosophila visual system, Wong et al. found that inhibiting activation of autophagy through an mTOR signal led to the death of photoreceptor cells in an age-dependent and light-dependent manner. Their research also revealed that autophagy could inhibit the turnover of the toxic rhodopsin–arrestin complexes produced during light transduction and play a protective role in retinal light damage [121]. Midorikawa et al. also confirmed the role of autophagy in rhodopsin-mediated light-induced photoreceptor cell death. They demonstrated that knocking out the genes of autophagy-essential components in Drosophila, such as Atg7 and Atg8, or the gene phosphatidylserine decarboxylase PSD essential for autophagosome formation caused light-dependent retinal degeneration. The degradation of rhodopsin was achieved through the interaction between autophagy/endosomal degradation pathways, and the autophagy signal pathway played an important role in light-induced photoreceptor degeneration [122].

Moreover, in mammalian cells, three known Atg8 homologues, GATE-16, GABARAP, and LC3B, are activated by Atg7, located in autophagosomes. Therefore, Atg7 plays a vital role in autophagy formation by activating the above two ubiquitin-like proteins. To investigate the function of Atg7 in photoreceptors of mouse system, Chen et al. created a rod photoreceptor in mice. The mice with Atg7 knockout showed the characteristics of photoreceptor degeneration, photoreceptor structure destruction, and outer retinal nuclear layer reduction when exposed to intensive light of 5000 lux for 2 hours [123].

The gene knockout technique proved that autophagy is closely related to the pathogenesis of light-induced retinopathy. Under photooxidative stress, autophagy may play an important role in maintaining retinal homeostasis independently or through cross-regulation with apoptosis.

### 7.4. Apoptosis

There are two principal pathways of apoptosis: the extrinsic pathway, activating intracellular apoptotic enzyme caspase through an extracellular signal [124], and the intrinsic pathway, activating caspase by releasing the apoptotic activator from mitochondria. The extrinsic apoptosis pathway is triggered by the ligation of tumor necrosis factor (TNF) and other death receptors on the cell surface, leading to the recruitment of the Fas-associated death domain protein (FADD) causing caspase-8 to undergo a self-proteolytic process and activation.

Once activated, caspase-8 can activate the downstream effector caspase-3 through direct proteolytic cleavage or indirect cleavage of BH3-only protein, resulting in classical caspase-dependent apoptosis [125]. The intrinsic apoptotic pathway is triggered by the release of mitochondrial cytochrome C due to a cell stress response or apoptosis signal [126]. As an apoptosis-inducing factor, cytochrome C can interact with Apaf-1, and the precursor of caspase-9 forms an apoptosome and then convenes and activates Caspase-3, which leads to caspase cascade reaction and apoptosis [125].

Short-wavelength light exposure, especially blue light exposure, can significantly induce apoptosis of retinal photoreceptor cells [63, 127]. Wu et al. showed that after continuously exposed Sprague Dawley rats to diffuse blue light (400–480 nm) of 0.64 W/m^2^ for 3–6 h, photoreceptor cells showed significant apoptosis characteristics: progressively concentrated and marginalized chromatin, contracted or curled, and fragmented nucleus, concentrated cytoplasm, formed apoptotic bodies. The damaged photoreceptors were quickly phagocytized within a few days.

With the TUNEL technique, they showed that many TUNEL positive labeled nuclei were in the outer nuclear layer of the retina where the photoreceptor is located and predominantly distributed in the upper temporal region of the retina which is most sensitive to blue light injury. Wu et al. found that the treatment of 3400 lux light exposure for 24 hours significantly reduced the thickness of ONL in rats and increased the number of apoptotic cells. However, intravitreal injection of the caspase-specific inhibitors zVAD and DEVD significantly blocked photoreceptor apoptosis [63]. This evidence suggests the caspase-dependent apoptotic pathway involves in light injury-induced death of photoreceptor cells.

### 7.5. Phagocytosis

Phagocytosis refers to one cell encapsulating another cell leading to death; its key feature is that inhibiting phagocytosis may prevent cell death [128, 129]. Phagocytosis mainly includes three steps: recognition, phagocytosis, and digestion. Phagocytes recognize target cells by “eat me” signals (such as phosphatidylserine or calreticulin) [128, 130]. The phagocytic phase of phagocytosis may be realized by the interaction of ligand UDP and P2Y6 receptors between the cells [131]. For example, glutamate-induced excitotoxic injury can lead to rapid and reversible phosphatidylserine exposure on the neuronal cell membrane surface, which is recognized and phagocytized by activated microglia. However, if there are no activated microglia, such neurons can survive for a long time. Another “eat me” signal is the exposure of calreticulin on the cell membrane surface caused by excessive endoplasmic reticulum stress. Phagocytes can cause phagocytosis through LRP (lipoprotein receptor-related protein) receptors [130]. An increasing amount of evidence shows that the death of neurons caused by phagocytosis is a common phenomenon in the process of development and pathology, and blocking phagocytosis or phagocytic signals may prevent cell death.

To investigate the role of phagocytes in blue light-induced retinopathy, Joly et al. established two mouse models, mice injected with green fluorescent protein-labeled bone marrow cells and green fluorescent protein-labeled microglia. They disclosed that blood-derived macrophages enter the eye through the optic nerve and ciliary body after blue light exposure and quickly migrate to the injured retinal region. Microglia residing in the retina are also activated rapidly, showing macrophage characteristics in the damaged area. Blood-derived and microglia-derived macrophages were involved in the phagocytosis of death photoreceptors, but the blood-retinal barrier was not significantly damaged. This evidence shows that phagocytosis is very important for the clearance of light-damaged photoreceptor cells [132].

## 8. Summary

**Figure 3:**
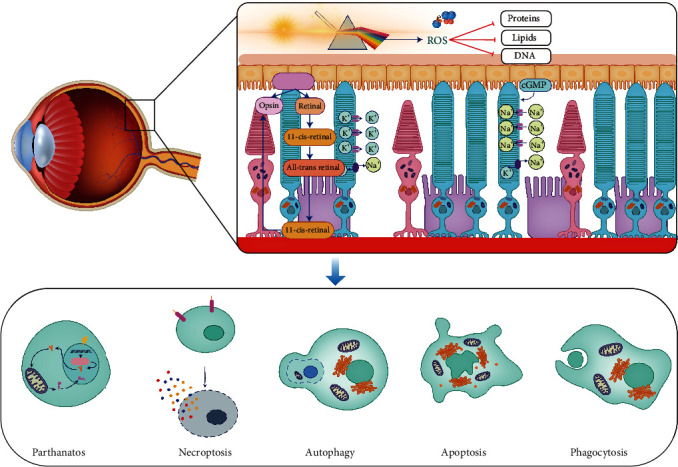
Molecular mechanism underlying light-induced injury to the retina. Photosensitive groups of photoreceptors can absorb the energy of photons after reaching the retina. In particular, the photons with short-wavelength contain high energy, which can break chemical bonds of macromolecule in cells and trigger photochemical reactions directly or indirectly. Photooxidation plays an important role in photochemical cascade reactions. Opsin involved in light transduction, mitochondria, and photosensitive macromolecules in cells can be important targets for generating reactive oxygen species. Excessive oxidative stress will damage the structure of cellular proteins, lipids, and DNA, which triggers the cell death cascade. The death pathways of parthanatos, necroptosis, apoptosis, autophagy, and phagocytosis are involved in light-induced death of photoreceptors, and these signal pathways may cross and convert each other (illustrated by Dr. XiaoLi Bao).

Photosensitive groups of photoreceptors can absorb the energy of photons after reaching the retina. Photons with a short wavelength contain high energy, which can break the chemical bonds of specific macromolecules in cells and trigger photochemical reactions directly or indirectly. Photooxidation plays an essential role in photochemical cascade reactions. Opsin involved in light transduction, mitochondria, and photosensitive macromolecules in cells can be important targets for generating reactive oxygen species. However, excessive oxidative stress in cells will further trigger cell death signals. Increasing experimental evidence shows that parthanatos, necroptosis, classical apoptosis, autophagy, and phagocytosis are involved in light-induced death of photoreceptors, and these signal pathways may cross and convert each other (Figure 3). Short-wavelength light, such as blue light, plays an essential role in light-induced retinal injury, so filtering out short-wavelength light may be an important strategy to prevent light injury-related retinal degenerative diseases.
